# Editorial: Innovative trends in vitreoretinal surgery: new technologies and smart ideas

**DOI:** 10.3389/fmed.2025.1602308

**Published:** 2025-05-28

**Authors:** Maurizio Mete, Antonio Moramarco, David Cordeiro Sousa

**Affiliations:** ^1^Ophthalmology Unit, Dipartimento di Scienze Mediche e Chirurgiche, Alma Mater Studiorum, University of Bologna, Bologna, Italy; ^2^Azienda Ospedaliero-Universitaria di Bologna, Bologna, Italy; ^3^Ophthalmology, Department of Surgery, University of Melbourne, Melbourne, VIC, Australia; ^4^Centre for Eye Research Australia, Royal Victorian Eye and Ear Hospital, Melbourne, VIC, Australia; ^5^Ophthalmology, University Hospital Geelong, Geelong, VIC, Australia

**Keywords:** vitreoretinal surgery, innovation, robotic surgery, imaging, teaching

In the dynamic field of ophthalmology, surgical innovation is continually reshaping treatment paradigms. Recent research spanning diverse patient populations—from pediatric cataract patients to individuals with complex retinal pathologies—demonstrates a shared theme: re-evaluation of traditional dogma and an embrace of newer, more adaptive approaches.

Take, for instance, the notion of “degeneration” in lamellar macular holes (LMH). The term has long implied irreversible retinal deterioration. However, Pertile et al. challenge this narrative through long-term data showing that “significant functional and microstructural improvements were observed after surgery” in both LMH and epiretinal membrane (ERM) foveoschisis, with no significant difference in outcomes between the groups (Pertile et al.). Their conclusion is clear: the healing potential evidenced postoperatively “questions the true ‘degenerative' nature of LMH” (Pertile et al.). Such findings urge the field to reconsider the fundamental definitions used in diagnostics.

Technological advancement also plays a pivotal role in improving pediatric care. Chan et al. document the successful application of an ultra-short 27G vitrectomy system in pediatric cataract surgery, noting a “significant short-term postoperative best-corrected visual acuity gain” in 60.5% of eyes (Chan et al.). While surgical complications occurred in over half of cases, the results reaffirm that modern instrumentation can reduce the invasiveness of procedures, improving outcomes even in vulnerable populations.

The utility of biologically derived materials is another frontier worth highlighting. Yang et al. explore the human amniotic membrane (hAM), now being used in posterior segment interventions. Known for its anti-inflammatory, anti-fibrotic, and neuroregenerative properties, hAM has been successfully employed in “repair[ing] photoreceptors, restor[ing] normal retinal structures, and clos[ing] abnormal structures in the optic disc” (Yang et al.). Its application to diseases such as retinal detachment and macular degeneration marks a significant step forward in tissue-based ophthalmic therapy.

Yet not all progress is technological. Sometimes, innovation arises from surgical creativity. Toro et al. present the T-shaped pars plana scleral incision as a novel technique for removing large intraocular foreign bodies (IOFBs), achieving a “100% removal rate” with no increase in iatrogenic trauma (Toro et al.). This seemingly small technical shift may represent a new standard in managing complex ocular trauma, minimizing surgical footprint while maximizing efficacy.

Novel materials have also been tested for intraoperative complications. Zou et al. report the successful use of cyanoacrylate glue to seal iatrogenic retinal breaks during surgery for stage 5 familial exudative vitreoretinopathy (FEVR), achieving anatomical stabilization in 88.9% of eyes (Zou et al.). While longer-term safety remains to be validated, this preliminary outcome offers hope for managing some of the most challenging pediatric retinal cases.

In Coats disease complicated by retinal cysts, Liang et al. provide a model of interdisciplinary and multimodal intervention. Their approach—endolaser photocoagulation combined with external cyst drainage and intravitreal anti-VEGF therapy—yielded full resolution of retinal cysts and stabilization in all 13 cases studied (Liang et al.). The study not only refines disease management but also underscores the necessity of personalized strategies for rare and aggressive retinal disorders.

Across these diverse studies, a common narrative emerges: surgical ophthalmology is in the midst of a quiet revolution. Whether by re-evaluating long-held assumptions, miniaturizing instrumentation, or deploying regenerative biomaterials, the field is moving beyond merely preserving vision toward truly restoring it. These advancements remind us that innovation is not always about inventing the entirely new—it is often about courageously rethinking what we already know.

